# Protocol of investigation into reporting quality of RCT abstracts on COVID-19 pursuant to CONSORT (CoCo study)—a review

**DOI:** 10.1186/s13063-021-05937-8

**Published:** 2021-12-24

**Authors:** Sabrina Tulka, Christine Baulig, Stephanie Knippschild

**Affiliations:** Chair of Medical Biometry and Epidemiology (IMBE), Faculty of Health, Witten/Herdecke University, Alfred Herrhausen Straße 50, 58448 Witten, Germany

**Keywords:** COVID-19, CONSORT, Reporting quality, RCT, Transparency, Coronavirus, SARS-CoV-2

## Abstract

**Background:**

In 2020, the COVID-19 pandemic developed into a global crisis, the enormity and urgency of which accelerated research activities in the field. At the same time, manuscripts describing these research projects underwent fast-track peer review procedures and were published in freely accessible formats. Although full texts about COVID-19 are currently available for free, abstracts continue to play a key role since they provide essential information and possibly a decision basis for therapies. Abstracts are particularly important in case the full texts are not free, not all reports have been published in English and in emergency situations when there is less time for comprehensive analysis of all full texts. It is therefore necessary to ensure that abstracts—as publications in miniature format—contain comprehensive and transparent information. The CONSORT statement for abstracts (CONSORT-A) offers guidelines to authors how to include all necessary information in an abstract.

Prior to the COVID-19 pandemic, the quality of reporting in medical research had already been the object of debate and criticism. The current crisis makes comprehensive documentation all the more important. Abstracts of COVID-19 RCTs should therefore report the criteria listed in the CONSORT-A statement fully and verifiably. The objective of this study is to check the completeness of abstracts of all COVID-19 RTCs published to date.

**Methods:**

Based on a literature search in PubMed, Embase and the Cochrane Library, all publications up to 29 October 2020 are identified and examined in terms of the subject matter (reported results from COVID-19 studies) and their study design (RTC). Subsequently, suitable publications are examined for completeness and quality of abstracts. The CONSORT checklist for RTC abstracts serves as a basis in this procedure. The primary endpoint of the study is the percentage of correctly implemented items of the CONSORT statement for abstracts. The frequency of correct reporting of each individual item is checked in a second step.

**Discussion:**

The study is expected to contribute to evaluating the reporting quality on COVID-19 studies, and specifically the completeness of abstracts of RTCs. It may thus support the assessment of current research into COVID-19.

**Trial registration:**

Registration was not required as the study investigated existing literature.

## Background

The quality and transparency of reporting in the field of medical research is frequently called into question and discussed extensively [1]. As early as 1996, the CONSORT group drew up a checklist and made it available to all authors of clinical studies as a guideline for transparent and comprehensive reporting. A detailed amendment referring to RCT abstracts (CONSORT-A statement) was added in 2008, since various studies revealed considerable shortcomings in the quality of abstracts [2]. Not all full texts are always freely accessible or available in English, so that abstracts play a key role in conveying pertinent information and often constitute the basis for therapy decisions. Nevertheless, the quality and completeness of RCT abstracts are frequently debated and challenged. Baulig et al. [3] found deficits in the reporting quality of abstracts from RCTs on age-related macular degeneration. Shaqman et al. [4] documented that out of 24 abstracts of RTCs exploring the effects of periodontal treatment on cardiovascular factors, not a single one offered exact information on the location of the trial, the patients included and methods of randomisation and blinding. Even criteria of essential importance for the interpretation of results were documented in only a small proportion of abstracts (results: 13%, number of patients analysed: 17%). These findings reveal massive deficits in terms of transparent and comprehensive reporting in RCT abstracts, with severe implications for a correct interpretation of study results.

In 2020, the COVID-19 pandemic developed into a global crisis, and teams of researchers worldwide started to investigate remedies, preventive measures, treatment strategies, vaccines, psychological effects and many further important aspects of a COVID-19 infection. In view of the enormous impact of the disease and the urgency of successful therapy options, papers on COVID-19 are being published in great numbers after accelerated peer review procedures. The aim is a fast and unobstructed transfer of knowledge. At the same time, this approach encourages the publication of insufficiently researched or poorly designed clinical studies. In the worst-case scenario, incorrect findings are published, undue conclusions are drawn and erroneous therapy decisions are made. In this context, publications (for example *Lancet* [5, 6] and *New England Journal of Medicine* [7]), ranked among the group of the five journals with the highest impact factors, had to be withdrawn early in the pandemic [8].

Full texts referring to COVID-19 have been made freely accessible as a rule. Nevertheless, abstracts continue to play a key role in the search for relevant literature and in deciding on promising therapy options. However, a certain percentage of publications remain inaccessible to many users despite open access because they were not published in English and for this reason are not accessible to all clinicians and scientists worldwide. In these cases, the abstract offers the only opportunity to obtain information. Moreover, acute situations like the COVID-19 pandemic do not always leave sufficient time to study all full texts and to make decisions in clinical practice on the basis of available evidence. For these reasons, an abstract should be drafted as a publication in miniature format to ensure complete and transparent reporting. Where the quality of reporting on medical (clinical) studies in particular is concerned, no compromises can be permitted. A standard with high quality is absolutely necessary and can be expected.

In view of the current relevance of the COVID-19 disease, the present study aims to assess the completeness of reporting in RCT abstracts on COVID-19.

## Methods/design

### Aim

The study aims to determine how many of the abstract criteria demanded by CONSORT are reported in published abstracts of RCTs on the treatment and prevention of COVID-19.

The study is conducted at the Chair of Medical Biometry and Epidemiology, Witten/Herdecke University. Researchers involved in the study are as follows:

Dr. Stephanie Knippschild

Dr. Sabrina Tulka, M.Sc.

Dr. Christine Baulig, M.Sc.

### Data

#### Search History

The literature search was conducted in 3 databases with the following keywords (29 October 2020):

(COVID-19 OR COVID) AND randomised controlled trial

Results of the search:

- PubMed (51 abstracts)
- Embase (38 abstracts)
- Cochrane – Trials (Central) (135 abstracts)

#### Selection of abstracts

**Fig. 1:**
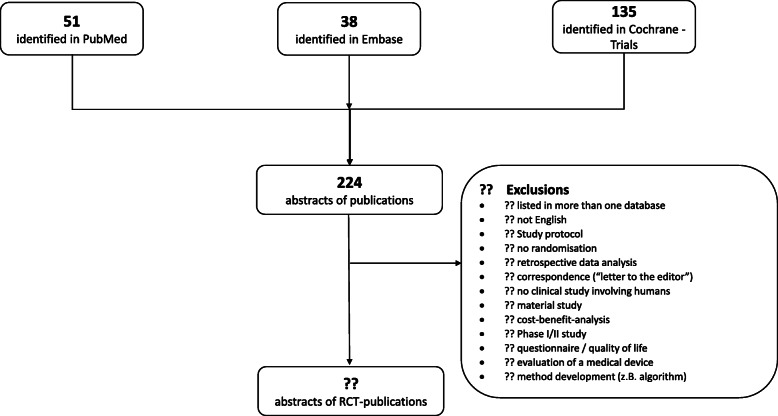
Flowchart of abstract selection. Overview of selection of suitable abstracts from RCT publications identified in the above-mentioned databases (PubMed, EMBASE, Cochrane)

*Inclusion and exclusion criteria for abstracts are as follows*:

All abstracts identified in the three databases will be examined in terms of suitability for inclusion in the study (see Fig. 1). These evaluation criteria were based on the CONSORT for abstracts, which is a recommendation for authors but can also be used to access the reporting quality in RCT abstracts.

All studies reporting findings from RCTs which explored therapies for the treatment or prevention of COVID-19 will be included (clinical studies involving humans (COVID-19 patients or healthy persons)).

Abstracts meeting at least one of the criteria listed below are excluded from the analysis:

- Published in a language other than English (a suitable abstract if published in English is also included if the full text is not available in English)
- Study protocols (study protocols of RCTs which otherwise meet requirements are excluded as well)
- Missing randomisation details/information
- Retrospective data analyses
- Correspondences (“letter to the editor”)
- Clinical studies not involving humans
- Material studies
- Cost/benefit analyses
- Questionnaire-based surveys/quality of life assessment
- Evaluation of medical devices
- Method development (e.g. algorithm)
- Further criteria observed during screening which preclude the possibility that a study can be an RCT exploring therapies for the treatment or prevention of a COVID-19 infection (randomised prospective controlled clinical study involving humans (COVID-19 patients or healthy persons)).

### Evaluation tool

Table 1 lists the individual criteria given in the CONSORT statement for abstracts and instructs how to determine whether the respective criterion is reported adequately. Evaluation is binary: 0 = not reported, not adequately reported, or incompletely reported; 1 = reported completely, pursuant to requirements.

**Table 1:**
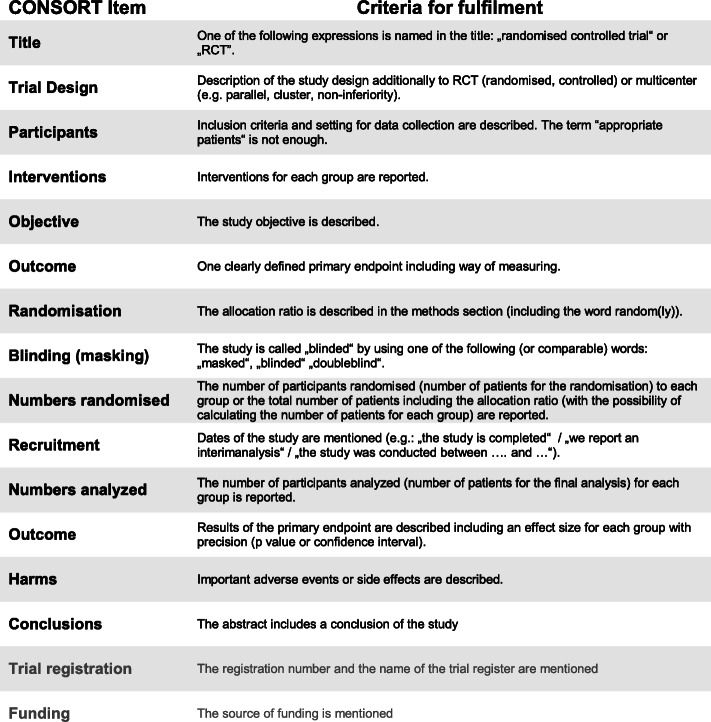
Evaluation tool: Basis for the evaluation of the criteria according to CONSORT-A. Trial registration and Funding will not be included (shaded in grey) as it depends on the journal if they are part of the abstract

Two raters (ST and SK) will carry out an evaluation on this basis and independently from each other. Subsequent to individual assessment, they will discuss their findings and in case of discrepancies settle on a final joint evaluation (consensus). Final and consented joint evaluations will be used to determine the results of the study. Cohen’s kappa as well as relative coincidence rates will be calculated to assess the agreement among raters (inter-rater reliability).

### Statistics and sample size calculation

#### Endpoints

The primary endpoint of the study will be the percentage of items from the CONSORT statement for abstracts which have been correctly implemented in an abstract. This percentage will be recorded for each abstract as relative frequency of CONSORT criteria fulfilled. Trial registration and funding will not be considered in this context since—depending on the publishing journal—they do not necessarily require this as part of the abstract.

Secondary endpoints will be the correct reporting of each single item. Emphasis will be placed on key criteria such as patient characteristics, results for each group, harms and the conclusion, since information on these factors may be considered of particular importance in the current COVID-19 pandemic.

Analysis of the studies will be exclusively on the basis of the abstracts without considering the full texts as the aim of this study is to check the simple and transparent reporting of all information in the CONSORT checklist for abstracts without evaluating the correctness of the reported results.

#### Sample size calculation

As we will evaluate all the abstracts available (see search history), a sample size calculation is not needed.

#### Evaluation strategy

The initial evaluation of the study will be solely descriptive. Results for the primary endpoint (proportion of correctly implemented criteria per study) will be presented as relative frequency per study. Results for the primary endpoint of the study will be displayed via calculation of measures of location (median, quartiles, minimum, maximum). Boxplots will be used in addition for graphic depiction. Absolute and relative frequencies of correct reporting on each individual item across all abstracts will be determined in order to establish the secondary endpoints.

Further collected data that characterise the abstracts will be analysed in this study and presented descriptively via frequency tables.

An additional explorative analysis of the primary endpoint is scheduled to be conducted via linear regression with the following determinants:

Multicenter study design (yes / no); trial registration (yes / no); origin of study (China / other than China—as China was the most frequent origin (43% of all results)); patient cohort (COVID-19 patients / healthy test persons), word count (number of words used in the abstract, determined via word count) and presentations of abstract (structured/unstructured).

We plan to examine a number of different factors potentially influencing the quality of the reporting. As it is impossible to say which factors will have an impact in advance, we have considered all additional study information available to us as possible influencing factors. In addition, we chose factors that were found as explanatory variables in other studies like the number of words used [3].

## Discussion

The study is scheduled to contribute to the assessment of reporting quality in randomised controlled clinical studies on COVID-19. Specifically, it serves to assess the completeness and transparency of information reported in the corresponding abstracts. The study aims to reveal possible deficits and thus to support high standards of reporting and provide assistance in presenting study results from current COVID-19 research.

## Trial status

Protocol version: 2; date: 02 November 2020

Day of literature search: 29 October 2020 (abstract collection is completed; abstracts will be screened for study eligibility).

## Data Availability

We give full access to collected data (our final and consented Excel table).

## Abbreviations

CONSORT: Consolidated Standards of Reporting Trials
CONSORT-A: Consolidated Standards of Reporting Trials – Extensions for Abstracts
COVID-19: Coronavirus-SARS-CoV-2
PubMed: Metadatabase containing references to medical publications from the entire field of biomedicine in the United States National Library of Medicine
RCT: Randomised controlled trial
EMBASE: Bibliographic database of biomedicine and pharmacology with published literature
